# Dissociative Disturbance in Hangul-Hanja Reading after a Left Posterior Occipital Lesion

**DOI:** 10.3233/BEN-2008-0211

**Published:** 2009-06-01

**Authors:** Key-Chung Park, Sung-Sang Yoon

**Affiliations:** Department of NeurologyKyung Hee Medical CenterKyung Hee University School of MedicineSeoulKorea

**Keywords:** Dyslexia, hangul, hanja, dissociation, posterior occipital area

## Abstract

Since the Korean language has two distinct writing systems, phonogram (Hangul) and ideogram (Hanja: Chinese characters), alexia can present with dissociative disturbances in reading between the two systems. A 74-year-old right-handed man presented with a prominent reading impairment in Hangul with agraphia of both Hangul and Hanja after a left posterior occipital- parietal lesion. He could not recognize single syllable words and nonwords in Hangul, and visual errors were predominant in both Hanja reading and the Korean Boston Naming Test. In addition, he had difficulties in visuoperceptual tests including Judgment of Line Orientation, Hierarchical Navon figures, and complex picture scanning. These findings are consistent with the hypothesis that Hangul reading impairment results from a general visual perceptual deficit. However, this assumption cannot explain why performance on visually complex Hanja was better than performance on visually simple Hanja in our patient. In addition, the patient did not demonstrate higher accuracy on Hanja characters with fewer strokes than on words with more strokes. Thus, we speculate that the left posterior occipital area may be specialized for Hangul letter identification in this patient. This case demonstrates that Hangul-Hanja reading dissociation impairment can occur after occipital-parietal lesions.

## 1. Introduction

Korean has two distinct writing systems, one using phonograms (Hangul) and the other ideograms (Hanja: Chinese characters), which correspond to Kana (phonogram) and Kanji (ideogram) in Japanese. There have been many studies showing dissociative disturbances in reading between Kanji and Kana. Anatomical lesions causing alexia for Kanji are usually located in the left posterior inferior temporal area, whereas alexia for Kana is reported after lesions in more diverse regions such as the posterior occipital lobe and the parietal lobe including the angular gyrus [5,6,9,10,12,14].

In contrast, selective alexia of Hangul or Hanja has rarely been reported. One case study reported a Korean patient with alexia and agraphia for Hanja but intact reading and writing for Hangul following a left posterior inferior temporal lobe infarction [3]. Another study showed a double dissociation of Hangul and Hanja reading and that the prominent alexia for Hangul was caused by an infarction in the left inferior parietal lobe, while the profound alexia for Hanja was caused by a cerebral hemorrhage in the left parietal lobe [4].

This present case describes a man who showed prominent reading impairment in Hangul with agraphia of Hangul and Hanja after a left posterior occipital lesion with parietal lobe involvement.

## 2. Case report

A 74-year-old right-handed male was admitted with sudden and subtle right side weakness upon waking. Two weeks prior, he had an episode of transient right side weakness. Past medical history was remarkable for pancreatic cancer with liver metastasis occurring 5 months prior and a 20-year history of diabetes and hypertension. The patient had been educated for 12 years

ISSN 0953-4180/08/$17.00 © 2008 – IOS Press and the authors. All rights reserved and had learned Hanja in elementary school under imperial Japanese rule. He had been fully independent and managing a clock store until his diagnosis of pancreatic cancer. His accounts were usually described in Hangul and Hanja. At admission, he was fully conscious and oriented. Neurologic and neuropsychologic examinations showed right homonymous hemianopsia, right arm numbness, mild right side weakness with right arm pronation and leg elevation drift, apraxia with body-part-as-object errors, and alexia and agraphia for Hangul.

A brain magnetic resonance imaging (MRI) scan 3 days after admission showed acute ischemic lesions affecting the left occipital and posterior parietal regions mainly under the lateral occipital gyri on T2-weighted and fluid-attenuated inversion recovery scans. In detail, the infarction involved the middle and inferior parts of the lateral occipital gyri as well as the left intraparietal sulcus, adjacent angular gyrus, and the left postcentral gyrus (Fig. 1). Brain single positron emission computed tomography revealed a perfusion defect in the left lateral occipital gyri and part of the parietal lobe.

The Korean-version of the Western Aphasia Battery (WAB) was administered 5 days after admission. Spontaneous speech (10/10) was fluent, and repetition (8.8/10) was relatively intact. He finished only the comprehension segment "yes-no" questions due to fatigue and abdominal discomfort, but answered all questions correctly (60/60). To evaluate his written naming ability in Hangul, the Korean-version of the Boston Naming Test (K-BNT) was administered; his score was 25/60, which is below the first percentile. Errors on the K-BNT included non-responses (17), visual errors (responding to a picture as another word that has visual resemblance or part of the whole image) (13), semantic errors (those associated semantically with the picture) (4), and phonological errors (one or more letters in Hangul words were substituted for other letters) (1). He could not perform the WAB reading and writing sections because of severe alexia and agraphia for Hangul and could not even read or write his name.

### 2.1. *Special tests*

To evaluate his Hangul reading ability in detail, single- and two-syllable reading tests consisting of primary school words were performed. He did not respond to two-syllable words (0/20), non-words (0/20), or single-syllable non-words (0/20), and he could read only a few single-syllable words (2/20). He also performed poorly in recognizing single unit digits (3/10). For Hanja reading, we presented 65 Chinese characters selected from 100 primary school basic characters. He read 48 of 65 Chinese characters correctly (correct response rate, 73.8%) and reported the character meaning; his mean reaction time was 7.65 sec per character. Errors in Hanja reading included non-responses (6), visual errors (changing one Hanja into the reading of another Hanja that has visual resemblance) (9) (e.g. 工 [goŋ], craft → 土 [to], soil/下 [ha], under → 子 [za], son/五 [o], five → 江 [gaŋ] river/ 兩 [ra:ŋ], amount → 雨 [u], rain/時 [si], time → 等 [du:ŋ] grade/ 王 [oaŋ], king → 羊 [a:ŋ], sheep/ 力 [rə:g], power → 右 [u], right/ 亡 [maŋ], be ruined → 己 [gi] body/ 老 [no], old → 孝 [ho:], obedience to parents) and unrelated responses (2). Hanja reading was followed by Hanja writing of the same 65 characters. However, when asked to write the character down after the meaning and sound were given, he gave up after a few trials because he could not recall the character imagery. Six age- and education-matched controls received the same Hanja reading tests and made errors in 0.5 of 65 characters, and the mean reaction time per character was 1.3 ± 0.2 (sec). To investigate the relationship between visual complexity and relative difficulty in Hanja reading, we estimated the number of incorrectly-read Hanja characters according to the number of strokes in the Hanja character lists. Of 45 Hanja characters with from 2 to 5 strokes (2 strokes: 1/4 incorrect, 3 strokes: 6/11 incorrect, 4 strokes: 3/12 incorrect, 5 strokes: 3/18 incorrect), the patient incorrectly read 13 Hanja characters (correct response rate: 70.1%). Of 20 Hanja characters with from 6 to 10 strokes (6 strokes: 2/7 incorrect, 7 strokes: 0/1 incorrect, 8 strokes: 1/7 incorrect, 9 strokes: 0/3 incorrect, 10 strokes: 1/2 incorrect), the patient incorrectly read 4 Hanja characters (correct response rate: 80%). Therefore, there was no apparent effect of visual complexity of Hanja characters on Hanja reading accuracy (chi-square test, $p = 0.452$).

To evaluate visuoperceptual ability, Benton Judgment of Line Orientation (JOLO), hierarchical Navon figures comprised of digits, and complex scenes such as the Cookie Theft picture were used. He could not complete the JOLO because of his inability to discriminate different angles of lines during the practice section (0/5). In addition, he was able to recognize only the smaller of the Navon figures and could only describe the complex picture in a piecemeal fashion. The patient refused follow-up examinations due to his poor general condition from pancreatic cancer.

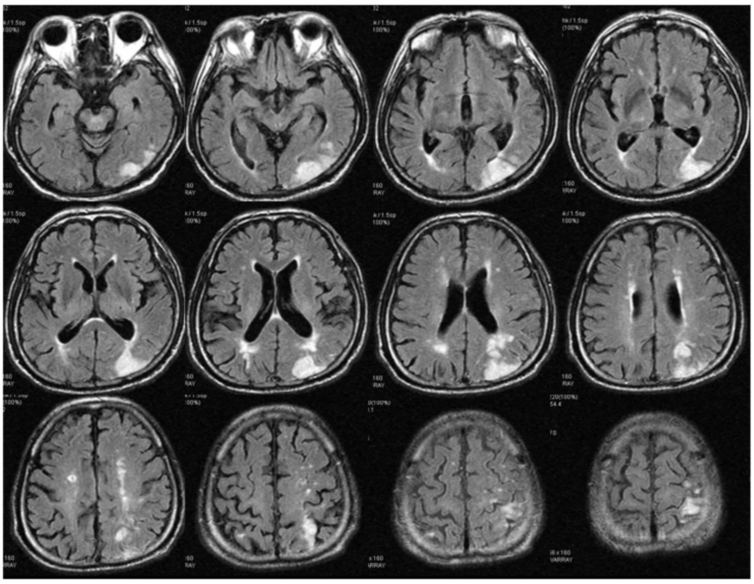

Fig. 1. Fluid-attenuated inversion recovery images obtained 3 days after admission show high signal intensities in the left posterior occipital region mainly under the middle and inferior lateral occipital gyri, as well as in the left intraparietal sulcus, adjacent angular gyrus, and left postcentral gyrus.

## 3. Discussion

The patient showed significant impairment in Hangul reading and severe difficulties in both Hangul and Hanja writing after a left posterior occipital lesion localized to the middle and inferior lateral occipital gyri, as well as to the intraparietal sulcus, adjacent angular gyrus and postcentral gyrus. A previous case study identified the posterior occipital lobe, including the lateral occipital gyri, as the anatomical substrate related to pure alexia for Kana in Japanese patients, which is consistent with the findings of our case [5,6,10]. Although one could argue that the angular gyrus lesion can cause alexia in our patient, several previous computed tomography or MRI studies have demonstrated that alexia does not consistently appear in lesions confined to the angular gyrus and that the involvement of the lateral occipital gyri posterior to the angular gyrus is likely to be related to reading impairment [9,15].

To explain the underlying mechanisms of Hangul-Hanja reading dissociation, it is essential to understand the different character features and reading processes between the two orthographic systems. Whereas Hangul is an alphabetical script, such as English or German, and can be characterized by grapheme-phoneme mapping in which larger units (syllables and words) are comprised of letters (e.g. ㄷ/d/- ㅏ/a/- ㄹ/l/ → 달 [dal], moon), Hanja is a logographic script, characterized by morpheme-syllable mapping in which a written character corresponds to a monosyllabic morpheme (e.g. 雨 [u], rain) [13]. Reading is performed via converting orthography into phonology, and two routes have been proposed for this process [2]. One is the lexical route, which is activated by recognizing a word-form as a whole, and the other is the phonology route, which converts word-form elements into corresponding sound elements. It is known that Hangul letters can be read by both routes and Hanja characters only via the lexical route [11]. Therefore, the predominant Hangul alexia in our patient implies a relatively selective injury to the phonological route. The ability to access the visual input lexicon or visual word form is preserved, but the early letter recognition stage is impaired.

The patient was unable to recognize even single-syllable words and nonwords in Hangul, and visual errors were predominant in Hanja reading and on the K-BNT. In addition, he had difficulties on several visuoperceptual tests including JOLO, hierarchical Navon figures and complex picture scanning. These findings may support the hypothesis that severe Hangul reading impairment is not confined to orthographic symbols but instead results from a general visuoperceptual problem [1]. However, this assumption cannot explain why the performance on the more visually complex Hanja was better in our patient. In addition, the patient did not demonstrate a better correct response ratio on Hanja letters with fewer strokes than on letters with more strokes. Thus, we consider that the left posterior occipital area may be specialized for Hangul letter identification in this patient. Sakurai et al's study provides some supportive evidence for our findings. Their positron emission tomography study reported that the lateral occipital gyri were activated predominantly during Kana reading, whereas the posterior inferior temporal area was affected during Kanji reading [7]. Another hypothesis suggests that the assembling process of two or four Hangul letters to recognize words would be impaired, but the patient did not perform differently in recognizing more visually-complex Hangul characters (e.g. ㅎ/ㅡ/ㄹ/ ㄱ → 흙) compared with Hangul characters composed of two letters (e.g. ㅈ/ㅏ → 자). However, we could not draw conclusions due to the lack of systematic and detailed analyses.

Previously, Kwon et al. described the hypothesis of double dissociation of Hangul and Hanja in Korean patients. However, the anatomical lesions that resulted in selective Hangul reading impairment and characteristics of language disturbances in their patient were different from those of our patient. The patient in the previous study had an ischemic lesion of the left postcentral gyrus, the inferior parietal lobule, and the posterior insula and showed Broca's type of aphasia [4]. Due to the Broca's aphasia, the reading ability of the patient was assessed by matching words and objects, and by choosing the matched one among five words after listening to the word; this differs from the usual procedure such as reading aloud. Although the patient showed dissociative performance on Hangul-Hanja reading in the set of matching words and objects, the result was not different in the set of choosing a matched one after listening to the word.

Although there are several reports of Kana alexia after posterior occipital lesions [5,6,10], Hangul has several different features from Kana. The shape of Hangul words is nonlinear and the composition of symbols is shaped into a square-like block whereas Kana is written in a serial order [13]. In addition, the usage of Hanja is much less in Korean language than the usage of Kanji in Japanese [4]. Different findings in our patient were also observed from the previous report of Kana alexia in Japanese. Although Hanja reading was better than Hangul, a mild Hanja alexia associated with visuoperceptual dysfunction was also observed. Severe agraphia for Hangul and Hanja was also accompanied by alexia. Parietal lobe involvement could be considered the cause for the mild Hanja alexia and the Hanja and Hangul agraphia. For writing impairments of Japanese letters, it has been reported that a lesion in the postcentral gyrus, the region surrounding the intraparietal sulcus, or the posterior inferior temporal gyrus can cause agraphia for both Kana and Kanji [6, 8]. The agraphia in our patient might be attributed to intraparietal sulcus and adjacent angular gyrus or postcentral gyrus involvement. Since the patient could not recall characters, involvement of the region surrounding the intraparietal sulcus may have a possible effect on agraphia [8].

This case study has several limitations. First, the acute ischemic lesion in our patient was not confined within the posterior occipital lesion and involved the posterior parietal region as well. Second, although there was a significant discrepancy of performance on Hangul-Hanja reading, the patient also shows mild Hanja alexia and severe difficulties in both Hangul and Hanja writing.

In conclusion, these findings support the hypothesis that the recognition of written Korean can be processed in different ways depending on two distinct writing systems, phonogram (Hangul) and ideogram (Hanja), and the left posterior occipital area may be specialized for Hangul letter identification. Further studies on patients with more circumscribed lesions are needed.

## Acknowledgment

This study was supported by a grant of the Korea Health 21 R & D Project, Ministry of Health & Welfare, Republic of Korea (A050079).
